# Molecular Dysfunctions of Mitochondria-Associated Endoplasmic Reticulum Contacts in Atherosclerosis

**DOI:** 10.1155/2021/2424509

**Published:** 2021-07-21

**Authors:** Xiaojiao Wang, Dan Luo, Sisi Wu

**Affiliations:** Core Facilities of West China Hospital, Sichuan University, Chengdu 610041, China

## Abstract

Atherosclerosis is a chronic lipid-driven inflammatory disease that results in the formation of lipid-rich and immune cell-rich plaques in the arterial wall, which has high morbidity and mortality in the world. The mechanism of atherosclerosis is still unclear now. Potential hypotheses involved in atherosclerosis are chronic inflammation theory, lipid percolation theory, mononuclear-macrophage theory, endothelial cell (EC) injury theory, and smooth muscle cell (SMC) mutation theory. Changes of phospholipids, glucose, critical proteins, etc. on mitochondria-associated endoplasmic reticulum membrane (MAM) can cause the progress of atherosclerosis. This review describes the structural and functional interaction between mitochondria and endoplasmic reticulum (ER) and explains the role of critical molecules in the structure of MAM during atherosclerosis.

## 1. Introduction

Atherosclerosis remains a leading cause of cardiovascular disease worldwide [1]. In the early stage of atherosclerosis, a large number of foam cells derived from macrophages and SMCs gather in the arterial intima lesions, forming dots or stripes of yellow fatty streaks [2]. As the disease progresses, fatty streaks will develop into fibrous plaques containing a large number of collagen fibers, foam cells, SMCs, extracellular matrix, and inflammatory cells [3]. In the end-stage of atherosclerosis, the deep cells of the fibrous plaque become necrotic and develop into atheromatous plaque (also named atheroma), with granulation tissue, a small number of lymphocytes, and foam cells [4]. Secondary changes such as plaque hemorrhage, rupture, thrombosis, calcification, aneurysm, and vascular lumen stenosis will occur during atherosclerosis [5]. The exact cause of atherosclerosis remains unclear. Hyperlipidemia, hypertension, smoking, diabetes, hyperinsulinemia, hypothyroidism, genetic factors, age, gender, obesity, and other factors are all considered risk factors for atherosclerosis [1]. The mechanism of atherosclerosis has not been well clarified. Cumulative low-density lipoprotein (LDL) arterial burden is a central determinant for the initiation and progression of atherosclerosis [6]. In the arterial intima, where plaque is easy to form, it is easy to retain and accumulate cholesterol-rich apolipoprotein B- (apoB-) containing lipoproteins (including LDL, their remnants, intermediate-density lipoprotein (IDL), and lipoprotein (a)) [1]. LDL could penetrate into the arterial intima exhibiting a strong atherosclerotic effect. With a weak antioxidant effect, LDL could further aggravate atherosclerosis after entering the lipid-rich atheromatous plaque. Various cardiovascular risk factors such as hyperlipidemia, diabetes, and vascular inflammation can induce endothelial cells damage and apoptosis during atherosclerosis [7]. Chronic inflammation driven by lipid is intimately involved in all stages of atherosclerosis progression. The infiltration and modification of the inner membrane of plasma-derived lipoproteins and their uptake are mainly from macrophages, which then form lipid-filled foam cells, triggering the formation of atherosclerotic lesions, while apoptotic cells and foam cells lack effective removal leading to the progression of the disease [8]. B cells, T cells, and some inflammatory factors also play a critical role in atherosclerosis [9–11]. Pan et al. combined SMC fate mapping and single-cell RNA sequencing of both mouse and human atherosclerotic plaques a novel cell state during SMC phenotypic switching and potential therapeutic targets for atherosclerosis [12]. Researches based on proteomics proves atherosclerotic samples had significant reductions in mitochondrial protein abundance [13]. Prolonged ER stress is an important cause of macrophage and possibly EC apoptosis in advanced lesions [14]. Additional ER stress–mediated proinflammatory effects in these cells may also affect early atherogenesis [14].

**Figure 1:**
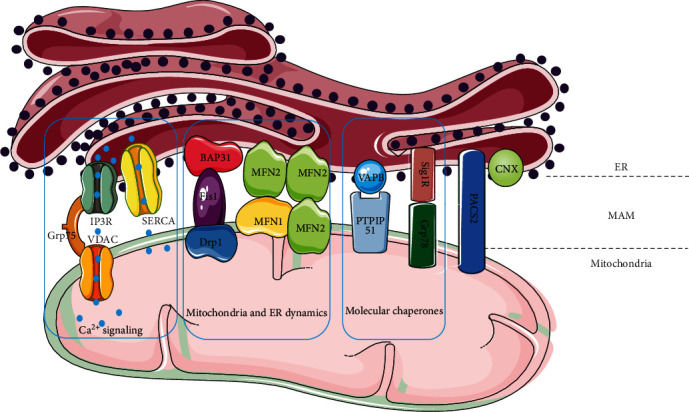
Convergence of signal transduction and metabolism at the MAM. The signal pathways that play the main biological functions on MAM including (1) calcium signaling (IP3R-Grp75-VDAC1 and SERCA-Ca^2+^) maintain the homeostasis of intracellular Ca^2+^and regulate the mitochondria and ER functions; (2) mitochondria and ER dynamics (MFNs and BAP31-Fis1-Drp1) regulate the fission and fusion of mitochondria thus regulating their functions; (3) MAM is the main place for the synthesis and transport of a variety of lipids; (4) molecular chaperones (VAPB-PTPIP51 and Sig1R-GRP78) participate in the secretion, folding, transport, and activation of proteins; (5) signal pathways such as PACS2-PSS1 can play other roles such as sorting and translocation.

As the site of oxidative phosphorylation, the double-membrane organelle, mitochondria provide a highly efficient route for eukaryotic cells to generate ATP from energy-rich molecules [15]. Mitochondria are not only the hub of biosynthesis, they can also balance redox equivalents and can also manage wastes such as ammonia, reactive oxygen species (ROS), and hydrogen sulfide [16]. The ER is a central intracellular organelle responsible for various key functions, including protein synthesis, folding, posttranslational modification and transportation; lipid and steroid synthesis; membrane synthesis and transportation; the metabolism of drugs and xenobiotics; the storage and release of intracellular Ca^2+^; the regulation of gene expression and energy metabolism; and the signaling to the nucleus, cytoplasm, mitochondria, and plasma membrane [17]. ER has a broad localization throughout the cell and forms direct physical contact with all other classes of membranous organelles, including the mitochondrial membrane [18]. ER and the mitochondrial membrane are closely connected in structure (called MAM, which has the properties of a lipid raft) and are highly related in function, regulating lipid metabolism, signaling transduction, the regulation of Ca^2+^ signaling, and the control of mitochondrial and ER biogenesis and intracellular transport [19]. The communication imbalance between ER and mitochondria will affect the function of MAM (Figure 1), which will lead to the occurrence of diseases.

Atherosclerosis has been associated with mitochondria dysfunction and damage, as well as ER stress [13, 20–22]. Studies have shown that the occurrence and development of atherosclerosis are closely related to the molecular changes on MAM [23]. This review is aimed at discussing the role of MAM structure and function in the pathophysiological process of atherosclerosis; it also clarifies how atherosclerosis-related proteins affect the structure and function of MAM.

## 2. Structural and Functional Proteins of MAMs Involved in Atherosclerosis

### 2.1. Calcium Signaling

#### 2.1.1. IP3R-Grp75-VDAC1

The voltage-dependent anion channel 1 (VDAC1) located in the outer mitochondrial membrane interacts with the inositol 1,4,5-triphosphate receptor (IP3R) on the ER through the glucose regulatory protein 75 (Grp75) (molecular chaperone), allowing Ca^2+^ from the ER is transferred to the mitochondria [24]. During the development of atherosclerosis, apoE may open the mitochondrial permeability transition pore (MPTP) through the interaction of apoE and VDAC1, thereby playing an important role in mitochondrial dysfunction induced by negatively charged LDL [25]. The secretion of apoE by macrophages occurs through protein kinase A (PKA) and Ca^2+^-dependent pathways along the microtubule network, and its specific expression can prevent atherosclerosis [26].

#### 2.1.2. SERCA-Ca2+

Sarco ER Ca^2+^-ATPase (SERCA), located in ER, is recognized as the main pump system that controls the cytoplasmic Ca^2+^ of muscle cells. This system could induce relaxation of Ca^2+^ from the cytosol. For each ATP hydrolyzed, SERCA pumps in two Ca^2+^ and releases less than four H^+^ into the cytoplasm, which indicates that the transport reaction is partially electrically induced [27]. Mitochondria can store large amounts of Ca^2+^ in its matrix. This feature only has a temporary effect, because if the mitochondria are forced to continuously accumulate Ca^2+^, they will not synthesize ATP, and ultimately deprive the Ca^2+^ pump of the energy necessary to remove Ca^2+^ from the cytosol, thus, triggering a vicious circle and aggravating Ca^2+^ production [27, 28]. Adenosine 5′-monophosphate- (AMP-) activated protein kinase (AMPK) acts as a physiological inhibitor of ER stress by maintaining SERCA activity and intracellular Ca^2+^ homeostasis, thereby alleviating the progression of atherosclerosis [29].

### 2.2. Mitochondria and ER Dynamics

#### 2.2.1. MFNs

Mitofusin (MFN), a membrane-bound dynamin-like protein named after its mitochondrial fusion activity, locating on the outer mitochondrial membrane (OMM) and mediates the merging of opposing OMMs in a GTP-dependent manner [30]. There are two types of MFNs in mammals, namely, MFN1 and MFN2, which have different roles in mediating OMM fusion. Both localize to OMM, while a small proportion of MFN2 is located at ER membranes. They form a complex in vitro in terms of structure and function, which has a greater fusion effect on isolated mitochondria [31]. MFN-mediated anchoring of mitochondria to transport proteins may contribute to mitochondrial motility [32]. MFN2 is a major (but not the only) protein tether linking mitochondria to ER/sarcoplasmic reticulum (SR) is substantial, perhaps incontrovertible [33, 34]. Mitochondrial-localized AIBP is related to MFN1 and MFN2 through its N-terminal domain and regulates their ubiquitination, thereby enhancing mitochondrial autophagy and participating in mitochondrial quality control, protecting macrophages from cell death in atherosclerosis [35]. Flavonoid binding protein 4 (FBP4) inhibits the expression of MFN1 in human aortic endothelial cells, but enhances the expression of dynamin-related protein1 (Drp1) and mitochondrial fission protein 1 (Fis1), suggesting mitochondrial fusion and fission dynamics are impaired, which induces oxidative damage of blood vessels and promotes the development of atherosclerosis [36].

#### 2.2.2. BAP31-Fis1-Drp1

There is an interaction between the resident ER protein B cell receptor-associated protein 31 (BAP31), which is involved in the initiation of mitochondrial fission process and the OMM mitochondrial fission 1(Fis1) protein [37]. In the fission cycle, Drp1 first binds to the mitochondrial fission factor (Mff) on the mitochondrial surface and then enters the complex containing Fis1 and BAP31 at the ER-mitochondrial interface, thereby inducing mitochondrial fission and downstream degradation [38]. Studies have confirmed that in the receptor for advanced glycation end products (RAGE) mice, increased mitochondrial fragmentation and increased expression of mitochondrial fission proteins Drp1 and Fis1 may promote the clearance of damaged mitochondria, thereby attenuating vascular oxidative stress and atherosclerosis caused by high-fat diet [39]. In human aortic endothelial cells induced by high glucose, mitochondrial fragmentation increased, Fis1 and Drp1 expression increased, indicating that increased mitochondrial fission may impair endothelial function by increasing reactive oxygen species, causing the process of atherosclerosis [40].

### 2.3. Lipid Metabolism

ER is the main site of lipid biosynthesis, but the synthesis and transportation of many kinds of lipids need to be completed at this specific site--MAM. For example, the unique pathway of intracellular phospholipid transmembrane transport occurs on MAM; MAM may serve as a site for the synthesis of cholesterol and neutral lipids; steroid production basically depends on the shuttle between sterols and mitochondria; MAM can also be used as a transport route for ceramide from ER into mitochondria [41]. MAM strictly regulates lipid transport between the ER and mitochondria. Long-chain acyl-CoA synthase (ACSL4 or FACL4) is a commonly used MAM marker. ACSL4 is located in MAM and plays an essential role in the regulation of mitochondrial fusion during cell steroid production [42]. Several enzymes related to cholesterol metabolism and transport have been found in MAM, including acetyl coenzyme A acetyltransferase 1 (ACAT1 or SOAT1) and acute regulatory proteins for steroid production (StAR) [43]. Caveolin-1 (Cav1) is an important regulator of cholesterol intracellular transport and membrane organization and is also identified as a key component of MAM. Loss of Cav1 gene in mice reduces the stability of ER mitochondrial contact sites and the accumulation of free cholesterol in MAM [44]. Macrophage cholesterol efflux capacity is dramatically reduced by inhibition of mitochondrial ATP synthase [45]. But how macrophage-derived foam cells control their energy metabolism is still unknown. The overexpression of StAR affects the lipid and inflammatory phenotypes of macrophages through cytochrome P450 27A1 (CYP27A1), liver X receptor *β* (LXR*β*) activation pathway and ATP-binding cassette transporter A1 (ABCA1/ABCG1) mRNA and protein induction pathway and promotes the efflux of cholesterol to apoA-I and/or high-density lipoprotein (HDL) [46, 47]. In addition, the injection of a viral vector expressing StAR into the tail vein of apoE (–/–) mice can reduce aortic lipids and atherosclerosis [48].

### 2.4. Molecular Chaperones

#### 2.4.1. VAPB-PTPIP51

Vesicle-associated membrane protein-associated protein B (VAPB) is an ER protein enriched in MAM, and protein tyrosine phosphatase interacting protein 51 (PTPIP51) is known as an OMM protein [49, 50]. VAPB was shown to bind to PTPIP51 to form at least some of the MAM tethers [51]. Regulating the expression of VAPB or PTPIP51 affects the Ca^2+^ exchange between the ER and mitochondria [50, 51]. Knockdown of VAPB or PTPIP51 by small interfering RNA (siRNA) decreases, while overexpression significantly increases ER-mitochondrial contact [51, 52]. A study showed that loosening ER-mitochondria contacts via loss of VAPB or PTPIP51 induces whereas tightening contacts by overexpression of VAPB or PTPIP51 impairs basal autophagy [53]. Existing studies have not found that VAPB or PTPIP51 are directly related to the progression of atherosclerosis. Oxidative stress, ER stress, and autophagy in macrophages are important causes of macrophage apoptosis in late atherosclerotic lesions, which can promote plaque necrosis, thereby exacerbating acute atherosclerotic cardiovascular events [54, 55]. It can be expected that future studies may find that autophagy caused by VAPB or PTPIP51 may be related to atherosclerosis caused by macrophage autophagy.

#### 2.4.2. Sig1R-Grp78

Sigma-1 receptor (Sig1R) is an ER molecular chaperone protein composed of 223 amino acids, which is highly expressed on MAM [56]. When the cell is stimulated by the outside, Sig1R can transfer from the ER to other parts of the cell. Sig1R is known to play an important role in many cellular activities such as ion channel activation, protein kinase A activation, neurotransmitter release, and inositol triphosphate receptor-mediated calcium transport from the ER to mitochondria [57]. The ER lumen and glucose regulatory protein 78/binding protein (Grp78 or BiP) is a member of the heat shock protein 70 family, a molecular chaperone and an ER stress sensor in MAM that aids in protein folding and secretion [58]. It folds the StAR transporting cholesterol for delivery to the OMM [59]. Under physiological conditions, Sig-1R forms a complex with Grp78 in MAM [60]. During ER stress, unfolded or misfolded proteins aggregate to dissociate and activate protein kinase R-like endoplasmic reticulum kinase (PERK), inositol-requiring enzyme 1(IRE1), activating Transcription Factor 6(ATF 6) and GRP78, which in turn triggers downstream signal transduction pathways [61, 62]. Unfold protein response (UPR) produces adaptive or proapoptotic responses based on the duration and/or intensity of stress [63]. The initial study showed that ER stress caused by increased Grp78 is related to the development of atherosclerotic lesions in apoE-/- mice [64]. Anti-GRP78 autoantibodies induce endothelial cell activation and accelerate the development of atherosclerotic lesions by activating the NF-*κ*B pathway, thereby inducing the expression of intercellular cell adhesion molecule-1 (ICAM-1) and vascular cell adhesion molecule-1 (VCAM-1) [65]. Recently, ER stress has been introduced into clinical applications: circulating GRP78/BiP is used as a sign of metabolic diseases and atherosclerosis [66].

### 2.5. Other Functions

#### 2.5.1. PACS2-PSS1

Phosphofurin acid cluster classification protein 2 (PACS2) is a multifunctional sorting protein which can interact with several cargo proteins and regulate their position in MAM [67]. PACS2 can transport Bim (Bcl-2-like protein 11) to lysosomes, participate in Ca^2+^ transfer from ER to mitochondria through IP3R positioning, and can also transport Bid (BH3 interaction domain) to mitochondria to control the induction of apoptosis [68, 69]. In mammalian cells, two different enzymes, phosphatidylserine synthase 1 (PSS1) and -2 (PSS2) in the MAM and the ER, perform de novo synthesis of phosphatidylserine (PS) [70]. Overexpression of PACS2 increased the level of PSS1 in MAM, indicating that PACS2 and PSS1 are functionally related [69]. A study found an increase in ER and mitochondria contacts as in PACS-2-associated MAMs upon stimulation with atherogenic lipids, while the disruption of MAM contacts by PACS-2 knockdown impaired mitophagosome formation and mitophagy, thus, potentiating VSMC apoptosis [23].

## 3. The MAM Hypothesis in the Pathophysiological Process of Atherosclerosis

### 3.1. The Role of MAM in the Development of Atherosclerosis Caused by Lipid Infiltration

On the one hand, LDL can penetrate the arterial intima and exhibit a strong atherosclerotic effect. On the other hand, LDL has a weak antioxidant effect. After entering the lipid-rich atherosclerotic plaque, it may further aggravate atherosclerosis. Researchers found that EC apoptosis induced by oxidized LDL (ox-LDL) is the initial step of atherosclerosis and is related to calcium overload [71]. The accumulation of Ox-LDL can increase cell apoptosis, accompanied by the increase of mitochondrial Ca^2+^, the loss of mitochondrial membrane potential (MMP), the production of ROS, and the release of cytochrome c [71]. PACS2 plays an important role in ox-LDL-induced EC apoptosis by regulating the formation of MAM and the increase of mitochondrial Ca^2+^, suggesting that PACS2 may be a promising target for atherosclerosis [71]. Recently, oxidized-LDL-mediated foamy macrophages were shown to exhibit an alternative mitochondrial metabolic switch from oxidative phosphorylation (OxPHOS) to superoxide production [72]. The content of MAM increases in atherosclerosis [23]. PACS2 has also been shown to be upregulated after atherosclerotic lipid load in VSMC [23].

### 3.2. The Role of MAM in the Development of Atherosclerosis Caused by Changes in VSMCs

The proliferation and migration of vascular SMCs (VSMCs) are one of the causes of atherosclerosis. VSMCs are the main cell type in all stages of atherosclerotic plaque. According to the “response to injury” and “vulnerable plaque” hypotheses, contractile VSMCs recruited from the media undergo phenotypic conversion to proliferative synthetic cells that generate extracellular matrix to form the fibrous cap and hence stabilize plaques [73]. The first important components that contribute to arterial stiffening are VSMCs, which not only regulate actomyosin interactions for contraction but mediate also mechanotransduction in cell-extracellular matrix (ECM) homeostasis [74]. Excessive saturated fatty acids have a damaging effect on VSMCs. A study proved that saturated fatty acids synthesized by the reaction of glycerol-3-phosphate acyltransferase 4 (GPAT4) at the contact site of *ω* and MAM can significantly inhibit the autophagic flux in VSMCs, thereby contributing to vascular calcification and apoptosis [75]. VSMC apoptosis accelerates atherogenesis and the progression of advanced lesions, leading to atherosclerotic plaque vulnerability and medial degeneration, suggesting MAM may be a new target to modulate VSMC fate and favor atherosclerotic plaque stability [23]. In addition, knocking out NgBR can cause MAM destruction and increase the phosphorylation of IPR3 through pAkt, is accompanied by mitochondrial dysfunction, including decreased Ca^2+^ respiration and mitochondrial superoxide, and increased mitochondrial membrane potential and HIF-1*α* nuclear localization, indicating that the dysregulation of NgBR promotes VSMC proliferation through the destruction of MAM and the increase of IPR3 phosphorylation, thereby contributing to reduce Ca^2+^ and mitochondrial damage [76].

### 3.3. The Role of MAM in the Development of Atherosclerosis Caused by Inflammation

Atherosclerosis is a chronic inflammation of the blood vessel wall. Inflammation is not only involved in the process of atherosclerosis but also causes complications such as thrombus and plaque rupture. Constituents of ox-LDL particles may induce inflammation and furnish neo-epitopes that stimulate humoral and adaptive immunity [77]. Many risk factors for atherosclerosis are involved in the activation of inflammatory pathways. In turn, inflammation can also change the function of arterial wall cells by driving atherosclerosis. These extravascular sites of inflammation can affect distant artery walls, as they release soluble inflammatory mediators such as cytokines that can activate cells in the intima [78, 79]. In macrophages from patients with atherosclerosis, mitochondria consumed more oxygen, generated more ATP, and built tight interorganelle connections with the ER, forming MAM [80]. The transfer of calcium through the MAM site continues to cause mitochondria to be overactive and depends on the inactivation of glycogen synthase kinase 3b (GSK3b), which controls the inflow of mitochondrial fuel, and therefore, represents a potential therapeutic target for anti-inflammatory therapy [80]. MAMs play a critical role in initiating inflammation by acting as an inflammatory platform [81]. The destruction of MAM in ECs attenuates mitochondrial damage, apoptosis, and inflammation and increases the release of NO [82]. Endothelial Drp1 silencing can prevent leukocyte adhesion and proinflammatory proteome induction, indicating that there may be cross-communication between typical inflammatory pathways and mitochondrial fission [83].

### 3.4. The Role of MAM in the Development of Atherosclerosis Caused by EC Injury

In a healthy state, when the lining of vascular endothelial cells is intact, SMCs can protect arteries from atherosclerosis and reduce endothelial inflammation; but when the lining of endothelial cells is damaged, changes in shear stress may directly affect the function of SMCs [84]. Under multiple overstimulations such as vascular shear stress, blood pressure, and cell mechanics, vascular endothelial cells induce endothelial cell proliferation or death by generating the degree of cytoskeleton deformation and reorganization allowed by the geometric shape of its environment [85, 86]. The injured endothelial cells secrete growth factors such as monocyte chemoattractant protein 1 (MCP1), platelet-derived growth factor (PDGF), and transforming growth factor *β* (TGF-*β*) and continuously ingest the lipids that enter the inner membrane and undergo oxidation to form foam cells. Downregulation of adipocyte PPAR*γ* can activate ER stress through the TLR4 pathway to upregulate the expression and secretion of the MCP1 gene, leading to enhanced chemotaxis of macrophages [87]. In atherosclerosis, pretreatment with tunicamycin (Tm), an ER stress inducer, significantly inhibited platelet-derived growth factor- (PDGF-) BB-induced VSMC proliferation and migration in a dose-dependent manner without causing significant apoptosis [88].

## 4. Conclusions

A large number of existing studies have described the structure and function of MAMs. MAM dysfunction has also received increasing attention from researchers on the occurrence and development of atherosclerosis. Any disturbance of calcium signaling, mitochondrial and ER dynamics, lipid metabolism, and molecular chaperones are all involved in the process of atherosclerosis. Atherosclerosis causes many morbidities and deaths worldwide, including most myocardial infarctions and many strokes, as well as disabling peripheral artery disease. This review describes the structural and functional interaction between mitochondria and ER and explains the role of critical molecules in the structure of MAM during atherosclerosis. It is expected that researchers can use MAM as one of the effective targets for the treatment of atherosclerosis in the future.

## Data Availability

All data included in this study are available upon request by contact with the corresponding author.
